# Recent advances of highly selective CDK4/6 inhibitors in breast cancer

**DOI:** 10.1186/s13045-017-0467-2

**Published:** 2017-04-24

**Authors:** Hanxiao Xu, Shengnan Yu, Qian Liu, Xun Yuan, Sridhar Mani, Richard G. Pestell, Kongming Wu

**Affiliations:** 1 Department of Oncology, Tongji Hospital of Tongji Medical College, Huazhong University of Science and Technology, 1095 Jiefang Avenue, Wuhan, 430030 People’s Republic of China; 2 Albert Einstein Cancer Center, Albert Einstein College of Medicine, New York, NY 10461 USA; 3 Pennsylvania Center for Cancer and Regenerative Medicine, Wynnewood, PA 19096 USA

**Keywords:** Breast cancer, CDK4/6 inhibitors, Palbociclib, Ribociclib, Abemaciclib, Safety, Treatment resistance

## Abstract

Uncontrolled cell division is the hallmark of cancers. Full understanding of cell cycle regulation would contribute to promising cancer therapies. In particular, cyclin-dependent kinases 4/6 (CDK4/6), which are pivotal drivers of cell proliferation by combination with cyclin D, draw more and more attention. Subsequently, extensive studies were carried out to explore drugs inhibiting CDK4/6 and assess the efficacy and safety of these drugs in cancer, especially breast cancer. Due to the insuperable adverse events and the less activity observed in vivo, the drug development of the initial pan-CDK inhibitor flavopiridol was consequently discontinued, and then highly specific inhibitors were extensively researched and developed, including palbociclib (PD0332991), ribociclib (LEE011), and abemaciclib (LY2835219). Food and Drug Administration has approved palbociclib and ribociclib for the treatment of hormone receptor-positive, human epidermal growth factor receptor 2-negative advanced or metastatic breast cancer, and recent clinical trial data suggest that palbociclib significantly improved clinical outcome when combined with letrozole or fulvestrant. Besides, the favorable effects of abemaciclib on prolonging survival of breast cancer patients have also been observed in clinical trials both for single-agent and combination strategy. In this review, we outline the preclinical and clinical advancement of these three orally bioavailable and highly selective CDK4/6 inhibitors in breast cancer.

## Background

Breast cancer is the most common female tumor type and accounts for the leading cancer mortality in women worldwide [1]. In spite of the great achievement in diagnosis and treatment, breast cancer remains a significant global burden [1]. Sequencing of breast cancer genome and transcriptome has identified breast cancer as a malignant disease with vast heterogeneity which is categorized into five distinct molecular subtypes including luminal A, luminal B, human epidermal growth factor receptor 2 (HER2)-enriched, basal-like, and claudin-low [2]. Among these, luminal-type accounts for the most part of breast cancer and is characterized with the typical expression of estrogen receptor (ER) and/or progesterone receptor (PR), which can be effectively targeted with hormone therapy. However, some patients have intrinsic resistance or acquired tolerance to hormone or endocrine therapy, which hampers the survival prolongation of these patients. Basal-like breast cancer, which is characterized with comparatively aggressive phenotype and the absent status of ER, PR and HER2, still lacks efficient treatment strategy. Thus, novel effective therapies are urgently required for breast cancer population.

Disordered cell cycle regulation is induced by complex mechanisms including the functional imbalance of oncogene and anti-oncogene, and contributes to uncontrolled cell proliferation resulting in cancer formation [3–8]. The past decades have witnessed the great progress in developing novel effective therapies [9–12], especially through diverting tumor cells from a proliferation phenotype towards a non-division state. Among the emerging therapies, cyclin-dependent kinase 4/6 (CDK4/6) inhibitors are the most attractive findings. CDK4/6 coordinates the cell cycle progression by reversible combination with cyclin D [13], and the bipartite complex of these elements phosphorylates pivotal tumor suppressors and transcription factors, contributing to cell cycle progression [14–16]. The essential roles of CDK4/6 in cell cycle regulation make them effective targets for cancer therapeutic intervention, especially in breast cancer [17–19]. The orally highly selective inhibitors of CDK4/6 are currently under active investigation, including palbociclib (PD0332991), ribociclib (LEE011), and abemaciclib (LY2835219). Among these, palbociclib and ribociclib remarkably prolonged the progression-free survival (PFS) in combination with letrozole for patients with ER-positive/HER2-negative advanced breast cancer, and have gained accelerated approval from Food and Drug Administration (FDA) as initial endocrine-based therapy for these patients.

In this review, we will first elaborate the anti-tumor mechanisms of CDK4/6 inhibitors, and then trace the preclinical and clinical evidence of these three highly specific inhibitors. At last, we will discuss the possible future directions in this field.

## CDKs in cell cycle regulation

**Fig. 1:**
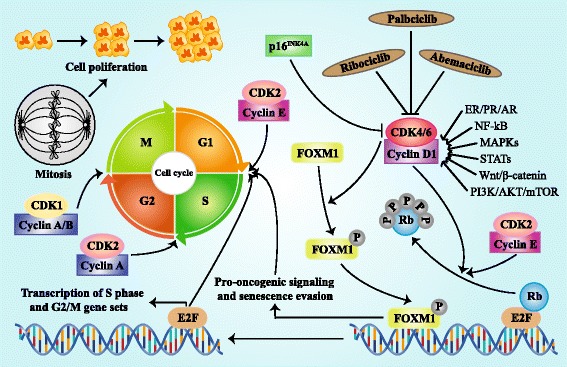
Regulation and function of CDK4/6 in cell cycle progression. Active complex of CDK4/6 and cyclin D phosphorylates and inactivates RB protein and then releases transcription factor E2F, triggering the up-regulation of E2F-responsive gene which promotes cell proliferation with cell cycle G1/S transition. The combination of CDK4/6 and cyclin D can also phosphorylates transcription factor FOXM1, resulting in the FOXM1-dependent expression of gene which protects cancer cells from cell cycle block. The kinase activity of CDK4/6 is suppressed by p16^INK4A^ and pharmacologic CDK4/6 inhibitors including palbociclib, ribociclib and abemaciclib. Cyclin D is regulated by multiple pathways such as ER/PR/AR, NF-kB, MAPKs, STATs, Wnt/β-catenin, and PI3K/AKT/mTOR. Besides, CDK2/cyclin E also participates in the RB phosphorylation. CDK2/cyclin A complex increases in stages S, G2, and M, while CDK1/Cyclin A/B complex mediates the transition from G2 to M stage

Cancer derives from uncontrolled cell division which results from the dysregulation of cell cycle progression including four stages of G1 (Gap phase 1), S phase (DNA synthesis), G2 (Gap phase 2), and M phase (mitosis) (Fig. 1). Cell cycle is monitored by a wide range of pathways including the retinoblastoma (RB)-E2F signaling [20]. RB, a well-known tumor suppressor, plays switching roles in cell cycle [20]. E2F is an evolutionarily conserved family of transcription factors, which functions in cell cycle control and contributes to tumor development [21]. The combination of RB and E2F makes E2F transcription modules in a suppressed state through inducing the recruitment of chromatin remodeling proteins, histone modifiers, and repressive chromatin marks, resulting in cell cycle block [22] (Fig. 1). The CDKs-RB axis is essential to cell cycle entry. CDK4/6 in combination with cyclin D, phosphorylates and inactivates RB [23], and then releases E2F, resulting in the recruitment of transcriptional activators, the alter transcription of genes involved during cell cycle process and subsequent G1-S block [22] (Fig. 1). Furthermore, the active combination of CDK4/6 and cyclin D is also involved in the phosphorylation of the cell proliferation-specific transcription factor forkhead box M1 (FOXM1), inducing the expression of genes which drive cell division and suppress cellular senescence in a FOXM1-dependent manner [24] (Fig. 1). However, the kinase activity of CDK4/6 is suppressed by p16^INK4A^ [25–27] (Fig. 1), and cyclin D is regulated by a complex network such as ER/PR/androgen receptor (AR), nuclear factor kB (NF-kB), mitogen activated protein kinases (MAPKs), signal transducers and activators of transcription (STATs), Wnt/β-catenin and phosphatidylinositol 3-kinase (PI3K)/AKT/mTOR [24] (Fig. 1).

In addition, the active combination of CDK2 and cyclin E also participates in the phosphorylation of RB (Fig. 1). Besides, CDK2 is accumulating and active in stages S, G2, and M in combination with cyclin E and cyclin A, respectively, while CDK1/cyclin A/B complex mediates the transition from G2 to M stage [22] (Fig. 1).

## CDK4/6 inhibitors

In consideration of the pivotal role of CDK4/6 in cell cycle progression, amounting studies have been conducted to suppress cancer cell proliferation through targeting the CDK4/6 signaling for effective cancer therapies during the past decades. Several CDK inhibitors have been explored for potential tumor treatment and assessed for pharmacokinetics, efficacy, and safety in many clinical trials.

**Fig. 2:**
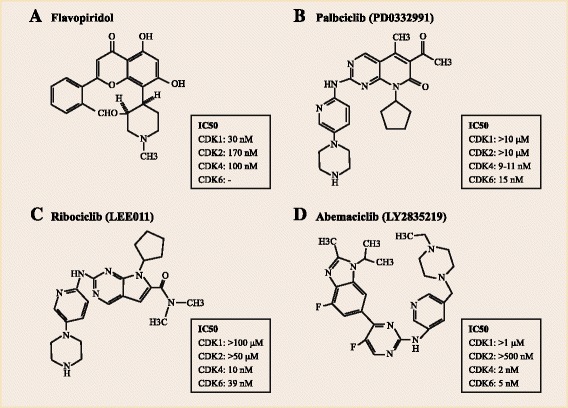
Chemical structures of CDK4/6 inhibitors. The chemical structures of the pan-CDK inhibitor **(a)** flavopiridol and the highly selective inhibitors including **(b)** palbociclib (PD0332991), **(c)** ribociclib (LEE011) and **(d)** abemaciclib (LY2835219) are shown. The reported half-maximal inhibitory concentration (IC_50_) values of these inhibitors are shown

Flavopiridol, also named as alvocidib developed by Sanofi-Aventis, is the most extensively investigated one among the first generation pan-CDK inhibitors, showing inhibitory effects on CDK1, CDK2, CDK4, CDK6, CDK7, and CDK9 [28–30], with half-maximal inhibitory concentration (IC_50_) values ranging from 20 to 170 nM (Fig. 2). In addition to cell cycle inhibition, flavopiridol-associated distal cellular effects also include apoptosis, transcriptional suppression, autophagy and endoplasmic reticulum stress [31–33], leading to several unacceptable high rates of dose-limiting toxicities, including neutropenia, hyperglycemia, cardiac, and pulmonary dysfunction [34]. The development of this non-selective compound was discontinued due to the low specificity for CDKs and narrow therapeutic window [17].

Subsequently, inhibitors highly selective for CDK4/6 were explored and developed, primarily through chemical screening and optimization via adding pyrido [2,3-d] pyrimidin-7-one compounds with a 2-amino pyridine side chain at the C2 position [35]. Chief among this new waves are palbociclib, ribociclib, and abemaciclib, all of which are orally administrated inhibitors and exhibit little or no suppression of other CDK activities at clinically achievable doses. Of these selective inhibitors, palbociclib is the most extensively investigated and has been evaluated in vitro and in vivo till recent times [36, 37]. It potently induces G1-S cell cycle block through blocking the phosphorylation of RB and related proteins and then down-regulating S-phase cyclins and mitotic regulatory genes as well as suppressing nucleotide biosynthesis and DNA replication [38, 39]. But its biological function has been limited because the anti-tumor effects of palbciclib is dependent on the presence of an active RB protein [36, 37]. Scientists at Eli Lilly and Novartis have also developed the parallel drugs ribociclib and abemaciclib, respectively [40–44].

The highly selective inhibitors are analogous to the pan-CDK inhibitor flavopiridol in structure but different in chemical function. The chemical structures of these four CDK4/6 inhibitors described above are showed in Fig. 2.

## Pre-clinical evidence

### In vitro an in vivo study

#### Palbociclib

Palbociclib halters the phosphorylation of RB protein and subsequently down-regulates E2F-targeted gene, which accounts for cell cycle arrest [11, 45]. Furthermore, palbociclib is also implicated to inactivate the transcription factor FOXM1 and its transcription targets which induce cell proliferation [45–48]. In addition to the well-known anti-proliferation effects, palbociclib also demonstrates dramatic promotion of epithelial-mesenchymal transition and tumor cell invasion [49]. Besides, this compound can also sensitize cancer cells to other regimes when in combination, such as chemotherapy [50] and ionizing radiation [51]. The potent anti-tumor effects of palbociclib have been observed in several tumor types, including T cell acute lymphoblastic leukemia (T-ALL) [45], hepatocellular carcinoma [39], neuroblastoma [40], renal cell carcinoma [52], myeloma [53], mantle cell lymphoma [54], pancreatic ductal adenocarcinoma [49], esophageal adenocarcinoma [55], medulloblastoma [51], melanoma [56], non-small cell lung carcinoma [57], and particularly in breast cancer [50, 58, 59].

Palbociclib was most extensively investigated in breast cancer. As a monotherapy, this compound potently blocks the cell cycle progression through inhibiting the hyperphosphorylation of RB protein in sensitive breast cancer cells [50]. Besides, when combined with tamoxifen or trastuzumab, it displayed obvious synergistic effects in ER+ cells and HER2-amplified cells, respectively [50]. Furthermore, this drug could re-sensitize the MCF7 cell line with acquired tamoxifen-resistance to this estrogen modulator [50]. Triple-negative breast cancer (TNBC), the most malignant molecular type and lack of well-established markers, appeared to be partially responsive to cytotoxic chemotherapy [58]. McClendon AK reported that combination therapy of palbociclib and doxorubicin displayed additive cytostatic effects in RB-proficient TNBC cells [58]. For combination therapy, palbociclib contributed to profound G1 block through targeting RB signaling, while doxorubicin led to cell accumulation in G2/M stage due to DNA damage response [58]. However, palbociclibin combination with doxorubicin ultimately resulted in recurrent subpopulation, which might be explained that palbociclib could antagonize the cell death induced by doxorubicin [58]. However, this issue might be resolved by an intermittent dosing schedule.

The efficacy of palbociclib have been evaluated in multiple animal models bearing different kinds of caner, including liver cancer [39], glioblastoma multiforme [60], pancreatic neuroendocrine tumors [61], gliomas [62, 63], colon carcinoma [36], especially breast cancer [36, 45, 64]. This compound led to growth arrest of xenograft tumors and prolonged survival of treated animals. Mice models with ERBB2-overexpressing breast carcinoma showed that CDK4/6 was pivotal to the maintenance of the disease [45]. Palbociclib administration delayed the progression of transplanted tumor through stimulating cancer cell senescence via strongly suppressing the phosphorylation of RB protein and subsequently down-regulating E2F-targeted genes, but did not have any effects on the apoptosis [45].

#### Ribociclib

Amounting in vitro studies have been carried out to investigate the function of ribociclib in cancers including leukemia [65], neuroblastoma [40, 66, 67], neuroendocrine tumors [68], liposarcoma [69], particularly breast cancer [70]. Combination of ribociclib and 3-phosphoinositide dependent protein kinase 1 (PDK1) inhibitor GSK2334470 potently suppressed the proliferation and increased apoptosis in ER-positive breast cancer cell lines [70].

The efficacy of ribociclib was also assessed in animal models bearing multiple cancer types including breast cancer [42, 70, 71], neuroblastoma [40, 66], and liposarcoma [69]. In consideration that the PI3K/Akt/mTOR signaling participates in the regulation of cyclin D and this pathway is activated in most breast tumors, the combination of ribociclib and PI3K/mTOR inhibitor alpelisib (BYL719) was investigated in breast carcinoma [20]. These two agents synergistically impaired breast cancer cell proliferation and tumor growth in mouse models in comparison with alpelisib incubation alone, and this combination was also effective in breast cancer models with alpelisib-resistance [42]. Similar phenomenon was also noted by Jansen VM that ribociclib in combination with alpelisib potently suppressed tumor progression of MCF7 mice xenografts [70]. In addition, there are also studies assessing the role of ribociclib in HER2-positive breast cancer. The results of the research by Goel S indicated that the complex of cyclin D1 and CDK4 played important roles in the resistance of HER2-positive breast cancer cells to the anti-HER2 therapy. In this context, the inhibition of CDK4/6 resensitized acquired-resistant xenograft mice models to HER2-targeted therapies and suppressed tumor recurrence in vivo [71].

#### Abemaciclib

Abemaciclib, the third parallel CDK4/6 inhibitor, has been assessed in several tumors up to now, including breast cancer [72], melanoma [73], and bladder cancer [74]. ABCB1 and ABCG2, which are important ATP-binding cassette (ABC) transporters, contribute to multidrug resistance in tumor chemotherapy through transporting anti-tumor drugs to the outside of cancer cells [72]. Abemaciclib treatment significantly sensitized ABCB1 or ABCG2 over-expressing cancer cells to respective chemotherapeutic drugs through impairing the “porter” roles of ABCB1 and ABCG2 in these transporters-amplified carcinoma cells, which implicated that abemaciclib could reverse the ABCB1 or ABCG2-induced multidrug resistance to some extent [72].

The efficacy of ribociclib was also assessed in animal models bearing multiple cancer types including breast cancer [75], melanoma [73], glioblastoma [76],and head and neck squamous cell carcinoma [77]. A representative ER-positive/HER2-negative human breast cancer T47D xenograft model displayed the obvious anti-tumor effects of single-agent treatment of abemaciclib [75].

### Therapy resistance

#### Palbociclib

The incoming resistance to targeted therapies is a major limitation to treatment efficacy [78]. According to the study of human breast cancer cell lines representing different molecular subtypes, ER-positive subtype (9/10) was the most responsible to the proliferation inhibition of palbociclib, followed by HER2-enriched type (10/16) [50].

Full understanding of the CDK4/6-RB-E2F pathway is pivotal to guiding the utilization of palbociclib treatment. Cancer cells that were intrinsically lacking of RB or harbored inactivation of this tumor suppressor were implicated to fail to effectively response to palbociclib treatment [36, 60, 79], probably due to the absence of the target for palbociclib in the RB-deficient tumor cell lines [38]. Nevertheless, this concept is not universal. The results from Dean JL reflected that knockdown of RB could facilitate only a partial resistance to CDK4/6 inhibition-induced cell cycle arrest, while overexpression of E2F was capable of leading to complete passby of CDK4/6 inhibition irrespective of RB status and palbociclib exposure [38]. This phenomenon might be explained by the elevated levels of p107 and p16^INK4A^ in breast cancer. It was observed that p107 accumulated and augmented in RB-deficient environment and was associated with the moderate suppression of E2F-regulated proteins with the treatment of palbociclib, compensating for the RB loss in multiple breast cancer cell lines [38] and hepatocellular carcinoma models [39]. Besides, CDK4/6 inhibitor p16^INK4A^ and CDKN2A gene were also implicated to play profound roles in drug resistance [56]. RB-deficient tumors tend to demonstrate extremely high expression of p16^INK4A^ [80]. P16^INK4A^-enriched breast cancer models displayed unresponsive status to palbociclib treatment because CDK4/6 had already been largely suppressed by the endogenous p16^INK4A^ [38]. P16^INK4A^ level plus RB status could be utilized together to predict the response of breast cancer patients to palbociclib therapy [79, 81]. Besides, the deletion or inactivation of CDKN2A gene also predicted sensitivity to palbociclib treatment [56].

#### Ribociclib

ER-positive breast cancer cell line was the well-established population which was most sensitive to CDK4/6 inhibitors. However, drug-resistant subgroup emerged after chronic ribociclib treatment with no cell cycle G1 block and the upregulation of pCDK2, cyclin A, cyclin D1, and cyclin E in comparison with the parental cells [70]. PDK1 was identified to be capable of sensitizing ER-positive MCF7 cells [70]. The exposure of PDK1 inhibitor GSK2334470 could eliminate the resistance of ribociclib-tolerance breast cancer cells to this compound, with remarkable reduction of pRB, pCDK2, cyclin A, cyclin D1, cyclin E, pS6, and pRSK2 [70]. These results indicated that PDK1 might be involved in the acquired resistance of ER-positive breast cancer to ribociclib treatment.

Rader J reported that most of the studied human neuroblastoma-derived cell lines were sensitive to the proliferation inhibition induced by ribociclib and the other models were completely resistant [40]. Furthermore, it was found that MYCN-enriched cell lines and sensitive cells mostly overlapped, while MYCN-nonamplified cells and resistant cells partially overlapped, indicating that MYCN level was positively associated with the sensitivity of neuroblastoma cells to ribociclib treatment [40].

In spite of the potent anti-proliferaion effects of short-term ribociclib exposure, chronic continuous treatment of ribociclibre-established cell cycle progression with the recovery of RB hyperphosphorylation at sites S780 and S807/811 as well as the up-regulation of cyclins D1, D2, and D3, implicating a compensatory retroaction promoting cell cycle progression [69].

## Clinical evidence

### Pharmacokinetics

Given the promising results from in vitro and in vivo studies, these three highly selective CDK4/6 inhibitors were further investigated for pharmacokinetics, efficacy, and safety in clinical trials. Reported clinical trials on palbociclib, ribociclib, and abemaciclib in breast cancer are listed in Table 1.

**Table 1:** Reported clinical trials with targeted CDK4/6 inhibitors in breast cancer

Breast tumor type	Phase	Dosage	Response rate	NCT	Ref
Palbociclib					
RB+ ABC *N* = 5	I	Administrated in six dose escalation cohorts (standard 3 + 3 design) MTD and RP2D: 125 mg	SD: 20% (1/5)	NCT 00141297	[84]
ER+/HER2- ABC *N* = 9	I	MTD: 125 mg; Palbociclib (125 mg QD, 3 weeks on/1 week off) plus letrozole (2.5 mg, continuous)	PR: 33% (2/6) SD: 33% (2/6)	NCT 01684215	[83]
ER+/HER2-ABC *N* = 165	II	Palbociclib (125 mg QD, 3 weeks on/1 week off) plus letrozole (2.5 mg, continuous)	PFS: 20.2 months for the palbociclib plus letrozole group and 10.2 months for the letrozole group (HR 0.488, 95%CI 0.319-0.748; one-sided *p* = 0.0004)	NCT 00721409	[88]
RB+ MBC *N* = 37	II	Palbociclib (125 mg QD, 3 weeks on/1 week off)	PR: 7% (2/28) SD: 50% (14/28) PFS: 3.8 months (1.9–5.8) for HR+/HER2- patients, 5.1 months (5.1–∞)for HR+/HER2+ patients, 1.5 months (0.62-∞) for HR-/HER2- patients, 4.5 months for HR+ patients, and 1.5 months for HR- patients	NCT 01037790	[87]
HR+/HER2-ABC *N* = 521	III	Palbociclib (125 mg QD, 3 weeks on/1 week off) plus fulvestrant (500 mg IM every 2 weeks For the first three injections and then every 4 weeks), or matching placebo plus fulvestrant	PFS: 9.2 months (95%CI, 7.5 to ∞) for palbociclib plus fulvestrant group and 3.8 months (95%CI, 3.5 to 5.5) for placebo plus fulvestrant group (HR: 0.42; 95%CI,0.32 to 0.56; *P* < 0.001)	NCT 01942135	[90]
ER+/HER2-ABC *N* = 666	III	Palbociclib (125 mg QD, 3 weeks on/1 week off) plus letrozole (2.5 mg, continuous)	PFS: 24.8 months for the palbociclib plus letrozole group and 14.5 months for the letrozole group (HR 0.488, 95%CI 0.319-0.748; one-sided *p* = 0.0004)	NCT 01740427	[89]
Ribociclib					
RB+ ABC *N* = 20	I	MTD: 900 mg QD for 3 weeks on/1 week off RDE: 600 mg QD For 3 weeks on/1 week off; Ribociclib: 600 mg QD 3 weeks on/1 week off or continuous	PR: 1 (1/20) (600 mg/day continuous)	NCT 01237236	[85]
HR+/HER2- RBC or MBC *N* = 668	III	Ribociclib (600 mg QD 3 weeks on/1 week off) plus letrozole (2.5 mg QD) or matching placeboplus letrozole.	PFS: ribociclib group versus placebo group (0.56;95%CI, 0.43to 0.72, *p* < 0.001); ribociclib group versus placebo group (63.0% (95%CI, 54.6 to 70.3) and 42.2% (95%CI,34.8 to 49.5) after 18 months, OR: ribociclib Group versus placebo group (52.7% and 37.1%)	NCT 01958021	[91]
Abemaciclib					
BC *N* = 66	I	Abemaciclib (200 mg Q12H continuous for 4 weeks)	PR: 31% in HR+ patients and none in HR- patients SD: 50% in HR+ patients and 33% in HR- patients	NCT 01394016	[75]
ER-/PR-/HER2 + BC *N* = 1	I	Abemaciclib (200 mg Q12H continuous for 4 weeks)	Tumor size decreases more than 30% from baseline	NCT 02014129	[93]

*Abbreviations: ABC* Advanced breast cancer, *CI* confidence interval, *ER+* Estrogen receptor-positive, *HER2-* Human epidermal growth factor receptor 2-negative, *HR* Hazard ratio, *HR* Hormone receptor, *IM* intramuscular injection, *MBC* Metastatic breast cancer, *MTD* Maximum tolerated dose, *N* Number of enrolled breast cancer patients, *NCT* National clinical trial, *OR* overall response, *PFS* Progression-free survival, *PR* Partial response, *QD* Once daily, *Q12H* Twice daily, *RB+* Retinoblastoma-positive, *RBC* Recurrent breast cancer, *RP2D* Recommended dose for phase II studies, *SD* Stable disease

Palboliclib is slowly absorbed and eliminated in cancer patients after orally administrated [82–84]. The phase I clinical study in Japanese patients with solid tumors was conducted to assess the pharmacokinetics of palbociclib in patients with solid tumors [83]. Its half-life was 23–26 h and there were no drug to drug interactions between letrozole and palbociclib in this study [83]. Besides, 125 mg once daily over 3 weeks on followed by 1 week off schedule was the maximum tolerated dose (MTD) and was recommended for both monotherapy and combination strategy in ER-positive/HER2-negative advanced breast cancer [83]. Another two phase I studies both enrolling different RB-positive solid tumor patients indicated that palbociclib was slowly absorbed with median time from oral dose to maximum plasma concentration (*T*_max_) 4.2 or 5.5 h and slowly eliminated with mean half-life 26.7 or 25.9 h, respectively [82, 84].

According to results of the phase II clinical trial conducted by Infante JR, MTD and recommended dose for expansion (RDE) of ribociclib were 900 and 600 mg daily of 3 weeks on and 1 week off over a 28-day schedule, respectively, based on the assessment on the safety and efficacy of the dose-escalation schedules [85]. Upon oral administration, ribociclib was absorbed with median *T*_max_ varying from 1 to 5 h [85]. The half-life of ribociclib was approximately 36 h [86] and the average effective half-life was approximately 32 h at the dose of 600 mg daily of 3/1 schedule [85]. During 17 days following oral dosing, plasma concentrations rapidly increased about two- to threefolds because of accumulation [85]. The level of LEQ803, the main active metabolite of ribociclib, was positively linked to the dose of the parent drug ribociclib [85].

Abemaciclib is absorbed slowly ranging from 4 to 6 h from oral dose to maximum plasma concentration [75]. Abemaciclib was widely eliminated and distributed, and the average terminal elimination half-life varied from 17.4 to 38.1 h without significant dose-dependent clearance [75]. The mean top of plasma concentration of patients with 150 and 200 mg twice daily treatment reached 249 and 298 ng/mL, respectively [75]. Also, the cerebrospinal fluid concentration of abemaciclib ranged from 2.2 to 14.7 nmol/L, which was beyond the dissociation constant of CDK4/cyclin D1 combination and was close to the unbound plasma concentrations [75].

### Single-agent strategies

According to the phase II clinical study on palbociclib monotherapy enrolling RB-positive advanced breast cancer patients including 31 patients with hormone receptor (HR)-positive/HER2-negative disease, 2 patients with HR-positive/HER2-positive disease, and 4 patients with HR-negative/HER2-negative disease, clinical benefit (CB) was noted in 7 individuals overall, all of which were HR-positive patients after the treatment of palbociclib at the recommonded dose 125 mg daily on the 3/1 schedule [87]. The median PFS of the HR-positive group versus HR-negative population was 4.5 and 1.5 months (*P* = 0.03), indicating ER-positive breast tumors were more responsive to palbociclib treatment than ER-negative disease [87]. Furthermore, the degree of previous endocrine therapy impacted the efficacy of palbociclib in breast cancer [87]. HR-positive patients who had received more than two lines of anti-hormone regimens enjoyed 3 months longer median PFS than patients who had received less than two lines of these regimens after palbociclib treatment [87]. However, prior therapy of cytotoxic drugs did not significantly affect the median PFS on palbociclib treatment [87].

A phase I dose-escalation clinical study on ribociclib for single-agent therapy, enrolling 132 Rb-positive solid tumors including 20 breast cancer cases, demonstrated that one breast cancer patient with positive status of CCND1 and ER achieved partial responses (PR) at the dose of 600 mg daily during continuous ribociclib treatment [85].

A phase I study was conducted by Patnaik A to assess the pharmacokinetic profile, efficacy, and safety of abemaciclib in cancer patients. In this study, a total of 225 patients were enrolled including breast cancer patients [75]. The efficacy of abemaciclib monotherapy was investigated in 47 breast cancer patients including the following three subtypes: HR-positive/HER2-positive (*N* = 11), HR-positive/HER2-negative (*N* = 25), and HR-negative (*N* = 9) [75]. The overall level of complete response (CR) plus PR plus stable disease (SD) was much higher in HR-positive population than HR-negative subgroup (80 versus 33%) [75]. About 31% achieved PR and 50% achieved SD among 36 HR-positive patients, while none had PR and 33% had SD in nine HR-negative individuals [75]. Furthermore, abemaciclib treatment improved median PFS to greater extent in HR-positive breast cancer patients (8.8 months) than in HR-negative patients (1.1 months) [75]. However, the HER2 status did not make significant difference in the effects of abemaciclib on PFS of HR-positive breast cancer population (7.2 versus 8.8 months) [75]. These data indicated that abemaciclib was highly effective in HR-positive breast cancer for single-agent therapy. In order to further investigate the efficacy and safety of abemaciclib monotherapy, a phase II study was conducted, which included 132 female patients bearing HR-positive/HER2-negative advanced or metastatic breast cancer with disease progression during both hormone therapy and 1 or 2 lines of chemotherapy [86]. Patients received abemaciclib treatment at the dose of 200 mg twice daily continuously. Of patients evaluable for response, the clinical benefit rate including CR, PR, and SD reached 42.4%, and the median PFS was 6 months [86].

### Combination strategies

#### Palbociclib

The phase II trial by Finn RS, enrolled 165 postmenopausal patients with advanced ER-positive/HER2-negative breast cancer who had not received any treatment for this malignant disease [88]. Patients were randomly assigned into two groups receiving continuous oral aromatase inhibitor letrozole 2.5 mg daily alone (*N* = 81) or letrozole 2.5 mg daily in combination with palbociclib 125 mg daily (*N* = 84) for 3 weeks on followed by 1 week off over 28-day schedule [88]. Median PFS of these patients was assessed, showing that palbociclib plus letrozole group enjoyed about 10 months longer to progression (20.2 months) than the letrozole group (10.2 months) (hazard ratio (HR) 0.488; 95% confidence interval (CI), 0.319–0.748; one-sided *P* = 0.0004) [88].

In order to further confirm and extend efficacy and safety data for palbociclib plus letrozole from the phase II study, Finn RS conducted a double-blind phase 3 study enrolling 666 postmenopausal patients with ER-positive/HER2-negative breast cancer who had not received any treatment for this deadly disease [89]. A total of 444 patients were randomly assigned to receive palbociclib plus letrozole and the other patients received matching placebo plus letrozole [89]. The primary endpoint PFS was evaluated and the results indicated that palbociclib in combination with letrozole dramatically prolonged PFS (24.8 months) in comparison with letrozole monotherapy (14.5 months) (HR 0.58; 95% CI, 0.46 to 0.72; *P* < 0.001) [89].

In addition to the combination of palbociclib and letrozole, the efficacy of palbociclib plus fulvestrant was also investigated in breast cancer patients. Turner NC carried out a phase III trial including a total of 521 patients with HR-positive/HER2-negative advanced breast cancer that progressed in the process of prior hormone therapy [90]. Patients were randomly grouped into two cohorts. Patients in cohort 1 (*N* = 347) recieved palbociclib (125 mg daily orally for 3/1 schedule) plus fulvestrant (500 mg intramuscularly every 3 weeks for the first three injections and then every 4 weeks), and cohort 2 population received matching placebo plus fulvestrant [90]. Results implicated that there was a clinical meaningful and statistically significant improvement in PFS in patients receiving palbciclib plus fulvestrant (9.2 months) in comparison with the placebo group (3.8 months) (HR 0.42; 95% CI, 0.32 to 0.56; *P* < 0.001) [90].

#### Ribociclib

A phase 3 trial carried out by Hortobagyi GN, enrolled a total of 668 female postmenopausal patients with HR-positive/HER2-negative recurrent or metastatic breast cancer who had not receive systemic therapy for this advanced disease previously [91]. Among these patients, half were assigned to orally administer ribociclib (600 mg daily for 3 week on and 1 week off schedule) plus letrozole (2.5 mg daily), and the others were grouped to receive the treatment of matching placebo plus letrozole [91]. PFS was the primary endpoint of the study and overall response rate was one of the second endpoints [91]. The HR on PFS for ribociclib group versus placebo group was 0.56 (95% CI 0.43–0.72; *P* < 0.001). After 18 months, the PFS rate of ribociclib group was 63% (95% CI 54.6–70.3) and the placebo was 42.2% (95% CI 34.8–49.5) [91]. The overall response rates of ribociclib group and placebo group were 52.7 and 37.1%, respectively (*P* < 0.001) [91].

In consideration of the synergistic anti-tumor effects of ribociclib in combination with alpelisib in vitro and in vivo, this combination was also assessed in clinical trials. The phase Ib/2 study was conducted by Bardia A to investigate the safety and efficacy of triple combination of ribociclib plus exemestane and everolimus, enrolling 70 postmenopausal ER-positive/HER2-negative advanced breast cancer patients with letrozole- or anastrozole-resistance [86]. The recommended dose for phase II studies (RP2D) was established at 300 mg daily over 3/1 schedule plus 2.5 mg daily continously plus 25 mg daily continuously for ribociclib, everolimus and exemestane, respectively. Of 55 patients evaluable for response, 1 patient achieved CR, 5 patients achieved PR, and 26 individuals achieved SD [86].

Another phase Ib/2 trial assessed the triple combination of ribociclib plus letrozole and alpelisib in ER-positive/HER2-negative breast cancer patients. In this study, patients were grouped in three cohorts: cohort 1 (*N* = 41) for ribociclib plus letrozole, cohort 2 (*N* = 21) for alpelisib plus letrozole, and cohort (*N* = 36) for ribociclib plus alpelisib [86]. RP2D was established at 300 mg daily over 3 weeks on followed by 1 week off schedule plus 200 mg daily continuously plus 25 mg daily continuously for ribociclib, alpelisib, and letrozole, respectively [86]. Among 27 patients evaluable for response, PR was observed in 6 patients including 2 in confirmed PR and 4 in unconfirmed PR, and SD was also noted in 6 patients [86].

#### Abemaciclib

The phase I study carried out by Patnaik A which was described previously in this review, not only assessed the efficacy of abemaciclib monotherapy but also investigated the combination of abemaciclib and the antiestrogen agent fulvestrant [75]. In this study, 19 HR-positive breast cancer patients were enrolled in this cohort. Among this population, four patients (21%) achieved PR. The clinical benefit rate was 63%, which was similar to that of the single-agent strategy [75]. A phase Ib study showed that the disease control rate of CR, PR and SD was 67% in 36 breast cancer patients with the treatment of abemaciclib plus letrozole or abemaciclib plus anastrozole, and 75% of 16 patients with abemaciclib plus tamoxifen [86].

### Safety profile

The management of drug-related adverse events is a pivotal aspect of treatment. Reported clinical adverse events caused by palbociclib include neutropenia, leucopenia, fatigue, pulmonary embolism, back pain, and diarrhea. Among these, neutropeniais the primary toxicity of palbociclib [82–84, 87, 88, 92]. Previous study conducted by Flaherty KT enrolled 41 patients with distinct RB-positive solid tumors including melanoma, breast and other types, demonstrated that neutropenia is the only dose-limiting event and the most common non-hematologic adverse effects included fatigue, nausea, and diarrhea [84]. According to the phase 2 study by Finn RS, grades 3–4 neutropenia was noted in about half of advanced breast cancer patients treated with palbociclib plus letrozole, while in only 1% of patients treated with letrozole alone [88]. For leucopenia and fatigue, it was 19% versus none and four (4%) versus one (1%), respectively [88]. Furthermore, serious adverse events such as back pain, pulmonary embolism and diarrhea occurred in 2, 4, and 2% of palbociclib plus letrozole group, respectively [88]. But, febrile neutropenia or neutropenia-related infections were not observed among these patients during this study [88]. Also, according to the phase 3 study of 521 women with ER-positive/HER2-negative advanced or metastatic breast cancer, the adverse events were most commonly observed in palbociclib plus fulvetrant group in comparison with the placebo plus fulcestrant group, including neutropenia (62.0 versus 0.6%), leukopenia (25.2 versus 0.6%), anemia (2.6 versus 1.7%), thrombocytopenia (2.3 versus 0%), and fatigue (2.0 versus 1.2%) [90].

The safety of ribociclib was also assessed in clinical trials. According to the phase 3 clinical trial by Hortobagyi GN, common grade 3 or 4 adverse events were neutropenia (59.3% in the ribociclib group and 0.9% in the placebo group) and leukopenia (21.0 versus 0.6%) [91]. Infante JR reported that neutropenia and thrombocytopenia were the most common dose limiting toxicities (DLT) according to the MTD determination on seventy patients after cycle 1 treatment [85]. The most common hematologic adverse events were treatment-related neutropenia, leukopenia, thrombocytopenia and anemia, and the most common non-hematologic treatment-related adverse events were fatigue, nausea, and vomiting for all grades [85]. Approximately 9% of patients treated at 600 mg daily of 3 weeks on followed by 1 week off schedule experienced treatment-related asymptomatic QTcF prolongation, but grade 3/4 asymptomatic QTcF prolongation only occurred at the dose of more than 900 mg daily [85]. It was reversible and parallel with the maximal plasma concentration kinetics [85].

Abemaciclib treatment represents a distinct toxicity profile. In contrast to palbociclib and ribociclib, the DLT of abemaciclib was fatigue and this agent produced relatively less neutropenia might be due to the higher specific selectivity of this agent for CDK4 than for CDK6 [86]. The clinical study of a total of 225 patients with multiple cancer types showed that abemaciclib treatment related adverse events of all grades included diarrhea, nausea, fatigue, vomiting, leukopenia, thrombocytopenia, neutropenia, anemia, anorexia, increased creatinine, and weight loss [75]. The most common adverse events caused by abemaciclib treatment included fatigue and the gastrointestinal, renal and hematopoietic systems [75]. Grade 3 fatigue was DLT and the MTD was 200 mg every 12 h [75]. At 200 mg twice daily, one out of seven patients experienced DLT of grade 3 fatigue, and 275 mg twice daily endowed two out of three patients with the DLT of grade 3 fatigue [75]. According to a phase 1 clinical trial of 12 cancer patients demonstrated that diarrhea was the most common treatment–emergent adverse event and it could be managed to have no effects on the continuation of abemaciclib treatment at the dose of 200 mg twice daily [93].

## Conclusions

Taken together, oral highly selective CDK inhibitors, including palbociclib, ribociclib, and abemaciclib, represent an important therapeutic advancement in breast oncology. Apart from the clinical success of palbociclib and ribociclib, abemaciclib is in active investigation and the favorable effects of this agent on PFS of ER-positive/HER2-negative advanced breast cancer were observed in clinical trials. However, there are still some challenges in the optimization of CDK inhibitors in clinical practice. Firstly, there is still lack of predictive biomarkers to screen appropriate population who can benefit most from these agents. Selection of sensitive patients can improve the cost-effective ratio of these drugs. Although several studies have implied some potential candidates for sensitivity prediction such as the protein levels of RB and p16, further extensive clinical trials are urgently needed before applied as clinically useful biomarkers [94]. In consideration that liquid biopsy is a new technique for monitor tumor progression and treatment response [95–97], is it possible to identify potential biomarkers for predicting response to CDK inhibitors through analyzing circulating breast cancer cells or cell-free DNA? Secondly, aside from the already investigated combination strategies, whether CDK4/6 inhibitors in combination with other therapeutic regimens including chemotherapy, radiotherapy and immunecheckpoint inhibitors are also more effective than monotherapy is a problem, which is imperatively needed to be solved. For instance, there is some doubt whether palbociclib antagonizes the anti-tumor effects of cytotoxic chemotherapy and radiotherapy which function through killing cancer cells in cell cylce. Thirdly, triple-negative breast cancer, which is characterized with comparatively aggressive phenotype and the absent status of ER, PR, and HER2, is still lack of efficient treatment strategy. Previous study by Asghar U indicated that a subset of triple-negative breast cancer cells with expression of AR and the loss of cyclin E1 could be responsive to CDK4/6 inhibition [86]. Clinical trials are ongoing or in plans to address these questions.

## Abbreviations

ABC: ATP-binding cassette
AR: Androgen receptor
CB: Clinical benefit
CDK4/6: Cyclin-dependent kinases 4/6
CI: Confidence interval
CR: Complete response
DLT: Dose-limiting toxicities
ER: Estrogen receptor
FDA: Food and Drug Administration
FOXM1: Forkhead box M1
HER2: Human epidermal growth factor receptor 2
HR: Hormone receptor
HR: Hazard ratio
MAPKs: Mitogen-activated protein kinases
MTD: Maximum tolerated dose
NF-kB: Nuclear factor kB
PDK1: 3-phosphoinositide-dependent protein kinase 1
PFS: Progression-free survival
PI3K: Phosphatidylinositol 3-kinase
PR: Progesterone receptor
PR: Partial responses
RB: Retinoblastoma
RDE: Recommended dose for expansion
RP2D: Recommended dose for phase II studies
SD: Stable disease
STATs: Signal transducers and activators of transcription
T-ALL: T cell acute lymphoblastic leukemia
*T*_max_: Time from oral dose to maximum plasma concentration
TNBC: Triple-negative breast cancer
